# Supplementary material for: Placental OLAH Levels Are Altered in Fetal Growth Restriction, Preeclampsia and Models of Placental Dysfunction
Source: Antioxidants (Basel). 2022 Aug 27;11(9):1677. doi: 10.3390/antiox11091677 (PMC9495588; doi:10.3390/antiox11091677)
Supplement: Supplementary file 1 [file antioxidants-11-01677-s001.zip › antioxidants-1864821-supplementary.pdf]

Supplementary Material

Placental OLAH levels are altered in fetal growth restriction, preeclampsia and models of placental dysfunction

Authors: Natasha de Alwis, Sally Beard, Natalie K. Binder, Natasha Pritchard, Tu'uhevaha J. Kaitu'u-Lino, Susan P. Walker, Owen Stock, Katie Groom, Scott Petersen, Amanda Henry, Joanne M. Said, Sean Seeho, Stefan C. Kane, Lisa Hui, Stephen Tong, and Natalie J. Hannan

Contents

Supplementary Table S1. Patient characteristics of pregnancies complicated with fetal growth restriction with and without preeclampsia (collected as part of the FOX Study)....................... 2

Supplementary Table S2. Patient characteristics of samples used to assess the expression of OLAH over gestation. 3

Supplementary Table S3. Patient characteristics for placental samples collected from preterm, pathological pregnancies used to assess *OLAH* mRNA expression. 4

Supplementary Table S4. Patient characteristics for placental samples collected from preterm, pathological pregnancies used to assess OLAH protein production. 6

Figure S1. OLAH production in placental tissue collected from preterm pathological pregnancies. 7

Figure S2. Effect of silencing *OLAH* on cytotrophoblast cell survival.. 8

Figure S3. Effect of silencing cytotrophoblast *OLAH* on the expression and secretion of anti-angiogenic sFLT1 and angiogenic PGF under normoxic conditions (8% O_2)............................. 9

Figure S4. Effect of silencing *OLAH* on the expression of apoptosis, growth, inflammatory, oxidative stress and anti-oxidant genes under normoxic conditions (8% O_2).......................... 10

Supplementary Table S1. Patient characteristics of pregnancies complicated with fetal growth restriction with and without preeclampsia (collected as part of the FOX Study).

Characteristics	Normotensive (n=45)	Preeclampsia (n=69)
**Maternal age, years**	32 (27.5-35)	33 (30-37)
**Nulliparity**	24 (53%)	43 (62%)
**Body-mass index, kg/m²**	24 (21-25)	26.5 (22-32) *
**Smoking during pregnancy**	11 (24%)	4 (6%)
**Diabetes during pregnancy**	8 (18%)	7 (10%)
**Absent or reversed end diastolic flow in umbilical artery**	19 (42%)	27 (39%)
**Gestational age at delivery, weeks**	31.3 (30.05-32.35)	30 (28.3-32) *
**Birthweight, grams**	1090 (811.5-1295)	1094 (791.5-1301)
**Estimated fetal weight, grams**	1009 (746-1242)	1044 (71.3-1311)
**Male sex**	27 (60%)	42 (61%)
**Umbilical artery pH, median**	7.28 (7.25-7.31)	7.27 (7.22-7.3)
**Umbilical artery pH <7.2**	6 (13%)	8 (12%)
**Neonatal deaths within 42 days of birth**	0 (0%)	1 (1%)

Data are n (%),or median (IQR). Missing BMI data for n=3 normotensive samples and n=5 preeclamptic samples, and umbilical artery pH for n=1 sample in each group. Estimated fetal weight not available for n=4 normotensive and n=5 preeclamptic samples.*p<0.05.

Supplementary Table S2. Patient characteristics of samples used to assess the expression of OLAH over gestation.

	First trimester (n=11)	Second trimester (n=4)	Third trimester (n=10)
**Gestational age, weeks Median (IQR)**	9.1 (8.4-9.3) [a, c]	27.25 (24.95-28.2) [a, b]	39.1 (38.9-39.2) [b, c]
**Maternal age, years Median (range)**	25 (19-39) [c]	23.5 (19.5-26.75) [b]	33.5 (27-40) [b, c]
**Body mass index (kg/m^2) Median (IQR)**	24.8 (21.66-28.71)	40.90 (24-41)	26.61 (24.73-29.88)
**Parity, no. (%)**			
**0**	6 (54.5)	0 (0)	1 (10)
**1**	3 (27.3)	4 (100)	4 (40)
**≥2**	2 (18.2)	0 (0)	5 (50)
**Birthweight (g) Median (IQR)**	-	935 (736.3-1225) [b]	3415 (3085-3725) [b]

BMI data and maternal age unavailable for n=1 second trimester sample. All second and third trimester samples were delivered via caesarean section. Parity was not statistically analysed. [a]Significant difference between first trimester and second trimester characteristics. [b]Significant difference between second trimester and third trimester characteristics. [c]Significant difference between first trimester and third trimester characteristics.

Supplementary Table S3. Patient characteristics for placental samples collected from preterm, pathological pregnancies used to assess *OLAH* mRNA expression.

	**Preterm controls (n=10)**	**Preeclampsia (n=39)**	**Fetal growth restriction (n=14)**	**Preeclampsia + fetal growth restriction (n=10)**
**Maternal age, years Median (IQR)**	34 (26.5-37.5)	31 (27-36)	30 (25.3-33.5)	28.5 (22.75-31.25
**Gestational age at delivery, weeks Median (IQR)**	30 (29.4-31.58)	30 (28.1-31.4)	32.65 (30.85-34.0)*	28.95 (26.65-30.7)
**Maternal Body Mass Index (kg/m^2) Median (IQR)**	28.4 (24.0-30.0)	27 (23.95-37.08)	25.8 (18.75-29.5)	25 (22.8-30)
**Parity no. (%)**				
**0**	2 (20.0)	26 (66.7)	9 (64.3)	9 (90)
**1**	4 (40.0)	8 (20.5)	2 (14.3)	1 (10)
**≥2**	4 (40.0)	5 (12.8)	3 (21.4)	0 (0)
**Highest SBP prior to delivery (mmHg) Median (IQR)**	120 (110-126.3)	175 (160-185)****	120 (115-126.3)	165 (157.5-182.5)****
**Highest DBP prior to delivery (mmHg) Median (IQR)**	70 (67.5-76.25)	100 (100-110)****	76.5 (70-83.5)*	100 (90-112.5)****
**Birthweight (g) Median (IQR)**	1496 (1322-2011)	1127 (996-1435)*	1182 (973-1658)	623 (550-1041)***
**Male sex, no. (%)**	4 (40)	25 (64)	7 (50)	4 (40)

Maternal body mass index data unavailable for n=3 preterm controls, n=9 cases of preeclampsia (without growth restriction), n=1 case of fetal growth restriction, and n=3 cases of preeclampsia with fetal growth restriction. Birthweight data unavailable for a patient whose pregnancy was complicated by preeclampsia with fetal growth restriction. All samples

were collected after delivery by caesarean section. Statistical analysis was performed comparing all groups against the preterm control group. *$p<0.05$, ***$p<0.001$, ****$p<0.0001$

Supplementary Table S4. Patient characteristics for placental samples collected from preterm, pathological pregnancies used to assess OLAH protein production.

	Preterm controls (n=10)	Preeclampsia (n=6)	Fetal growth restriction (n=7)	Preeclampsia + fetal growth restriction (n=3)
**Maternal age, years Median (IQR)**	27 (23.75-33.75)	29.50 (25-31.5)	23 (19-32)	25 (21-26)
**Gestational age at delivery, weeks Median (IQR)**	30.3 (27.78-31.45)	32.1 (30.05-32.9)	31.3 (29.4-33)	30.8 (27.3-33)
**Maternal Body Mass Index (kg/m^2) Median (IQR)**	27.5 (24.25-35.23)	31.2 (25-37.5)	21 (18.5-29)	28.5 (27-30)
**Parity no. (%)**				
**0**	3 (30)	6 (100)	7 (100)	1 (33.3)
**1**	6 (60)	0 (0)	0 (0)	2 (66.6)
**≥2**	1 (10)	0 (0)	0 (0)	0
**Highest SBP prior to delivery (mmHg) Median (IQR)**	125 (111.5-130)	172.5 (155-185)***	125 (117-133)	160 (150-170)**
**Highest DBP prior to delivery (mmHg) Median (IQR)**	70 (68.75-78.5)	107.5 (92.5-112.5)***	80 (78-85)	90 (90-110)**
**Birthweight (g) Median (IQR)**	1492 (1231-1848)	1429 (1283-1547)	999 (858-1301)*	1008 (597-1393)
**Male sex, no. (%)**	4 (40)	2 (33.3)	1 (33.3)	3 (42.9)

Body mass index data unavailable for n=2 preterm control, n=1 preeclampsia (no fetal growth restriction), and n=1 preeclampsia with fetal growth restriction patients. All placental samples were collected at caesarean section. *$p<0.05$, **$p<0.01$, ***$p<0.001$

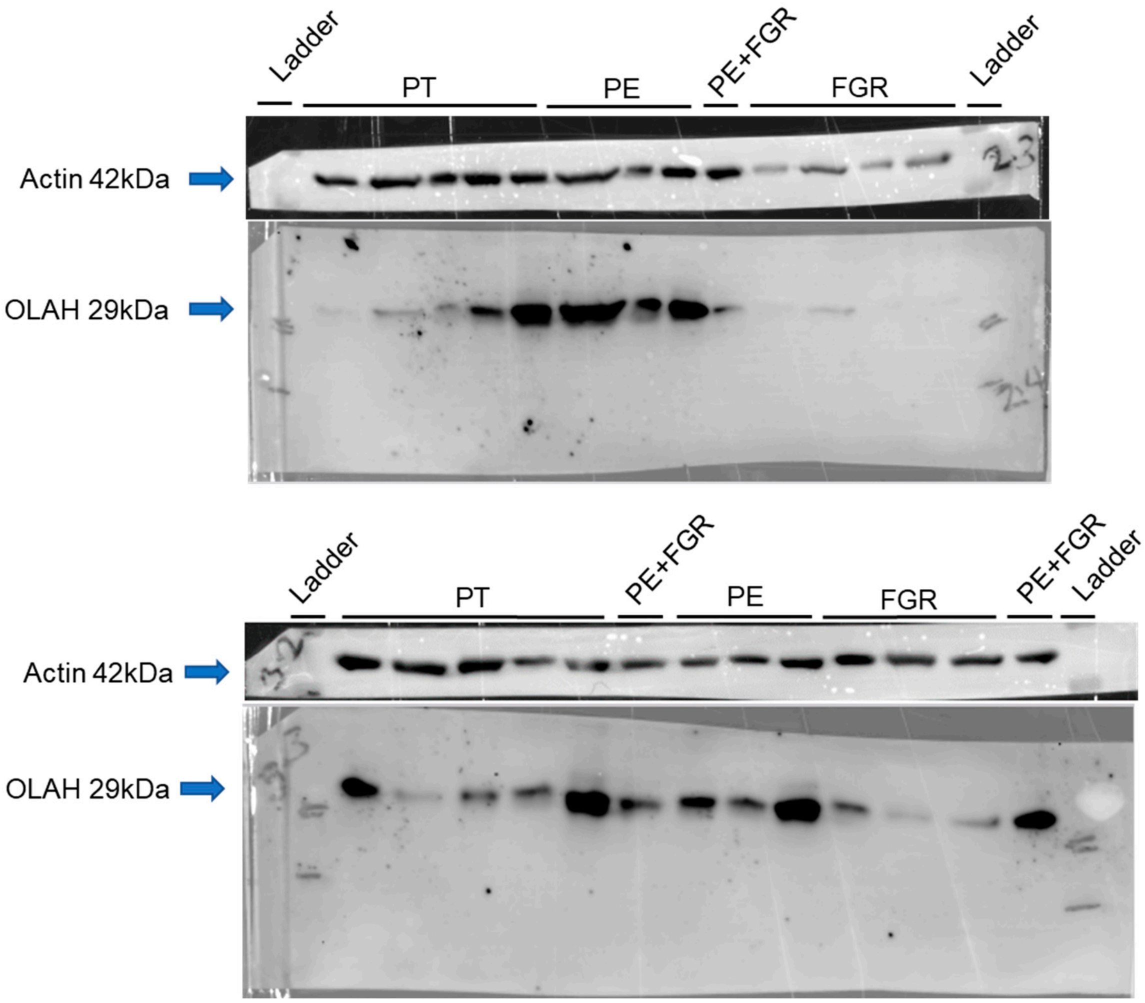

Supplementary Figure S1. OLAH production in placental tissue collected from preterm pathological pregnancies. Protein levels were determined via western blotting using placental tissue collected from pregnancies complicated by preterm preeclampsia without fetal growth restriction (PE), preeclampsia with fetal growth restriction (PE+FGR), fetal growth restriction alone (FGR), and preterm controls (PT). Corresponding densitometric analysis found in Figure 3.

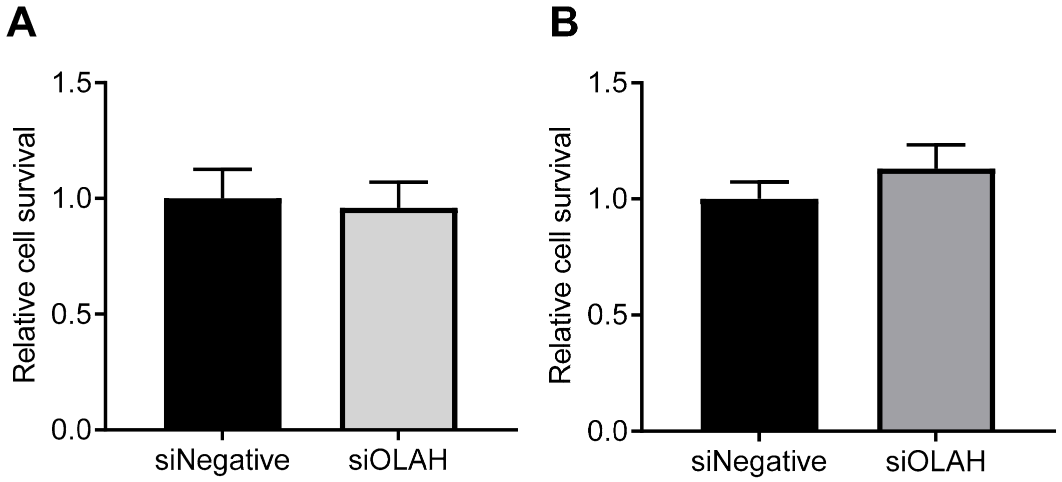

Supplementary Figure S2. Effect of silencing *OLAH* on cytotrophoblast cell survival. Cell survival was determined via MTS assay. Silencing *OLAH* did not significantly alter cell survival under either normoxic (A) or hypoxic (B) conditions. Data presented as relative change to control within each oxygen tension; mean ± SEM; n=3, each sample from a different patient.

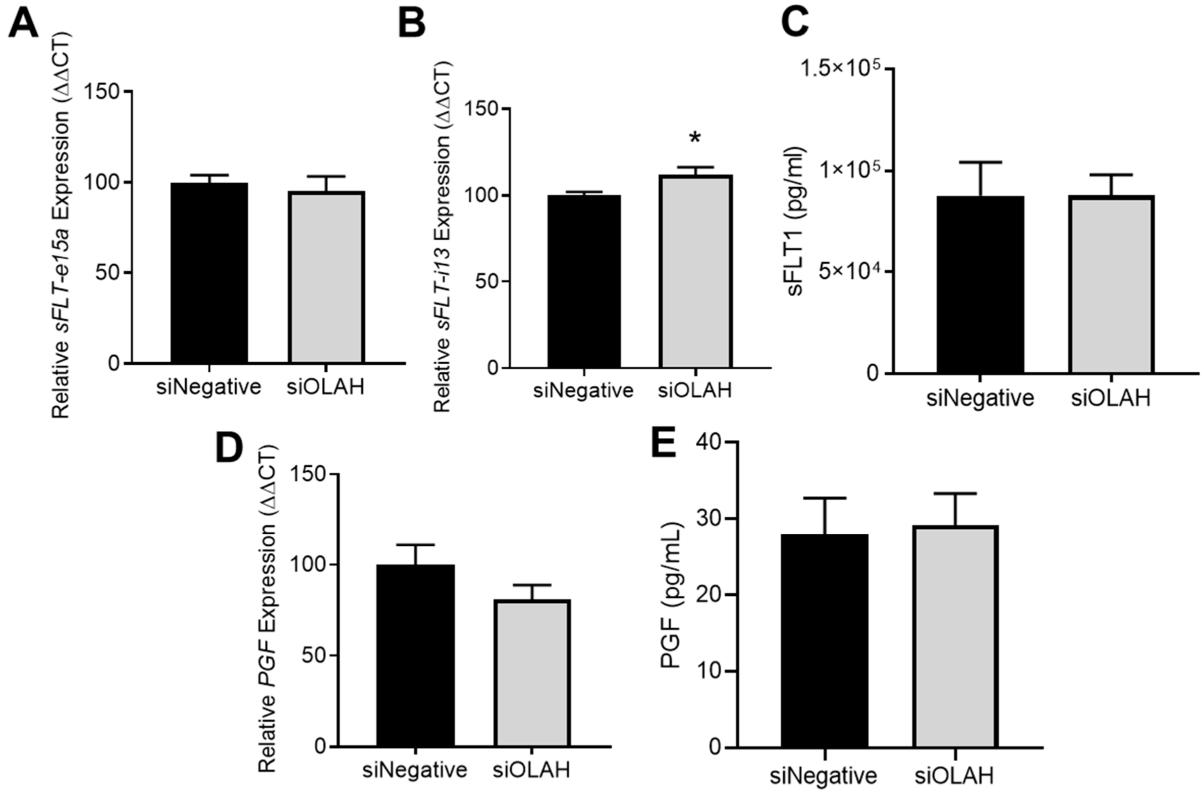

Supplementary Figure S3. Effect of silencing cytotrophoblast *OLAH* on the expression and secretion of anti-angiogenic sFLT1 and angiogenic PGF under normoxic conditions (8% O_2). Gene expression was assessed via qPCR and protein secretion via ELISA. Silencing *OLAH* in cytotrophoblast cells did not significantly alter the expression of sFLT1 isoform *e15a* (A), but significantly increased *i13* (B) expression, though sFLT1 secretion (C) was not significantly changed. Neither *PGF* expression (D) nor secretion (E) were altered with *OLAH* knockdown in normoxic conditions. Data presented as mean ± SEM; n=3, each sample from a different patient. *p<0.05.

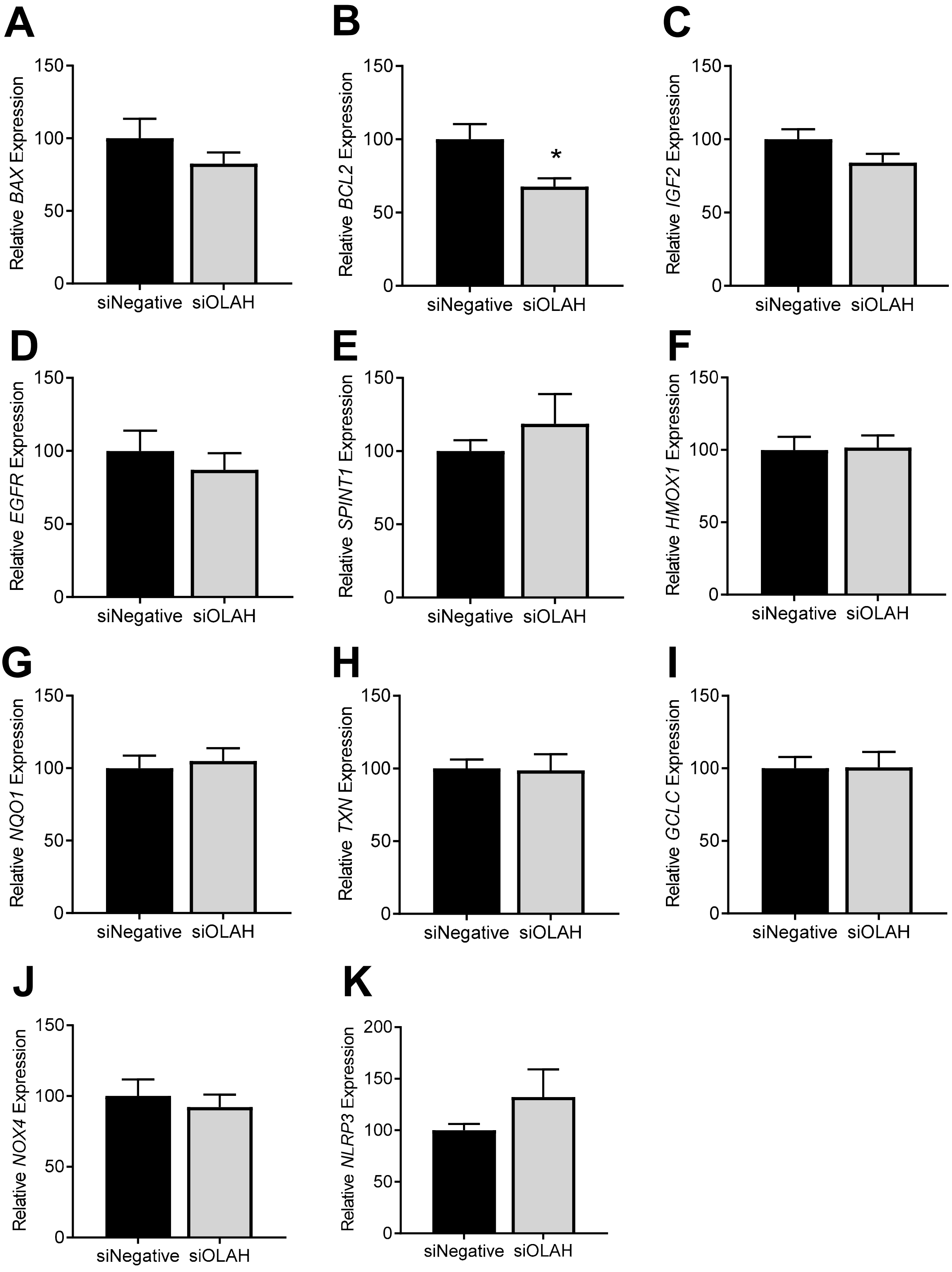

Supplementary Figure S4. Effect of silencing *OLAH* on the expression of apoptosis, growth, inflammatory, oxidative stress and anti-oxidant genes under normoxic conditions (8% O_2).

Silencing cytotrophoblast *OLAH* did not alter pro-apoptotic gene, *BAX,* but significantly reduced the expression of anti-apoptotic gene, *BCL2.* Silencing *OLAH* expression did not alter the expression of growth genes *IGF2, EGFR* or *SPINT1,* antioxidant genes *HMOX1, NQO1, TXN* or *GCLC,* oxidative stress gene *NOX4,* or inflammasome gene *NLRP3.* Results presented as relative change from control; mean ± SEM. n=3, each from a different patient. *$p<0.05$.
